# Nutritional Enhancement of Rice Noodles with Watermeal (*Wolffia globosa*)

**DOI:** 10.3390/foods14071096

**Published:** 2025-03-21

**Authors:** Nidthaya Seephua, Yu Liu, Hua Li, Apichaya Bunyatratchata, Onanong Phuseerit, Sirithon Siriamornpun

**Affiliations:** 1 Department of Food Technology and Nutrition, Faculty of Technology, Mahasarakham University, Kantarawichai, Maha Sarakham 44150, Thailand; 66010853001@msu.ac.th (N.S.); apichaya.b@msu.ac.th (A.B.); 2 Department of Cuisine and Nutrition, Yangzhou University, Yangzhou 225127, China; mx120231288@stu.yzu.edu.cn (Y.L.); lihua216@yzu.edu.cn (H.L.); 3 Key Laboratory of Chinese Cuisine Intangible Cultural Heritage Technology Inheritance, Ministry of Culture and Tourism, Yangzhou 225127, China; 4 Research Unit of Thai Food Innovation (TFI), Mahasarakham University, Kantarawichai, Maha Sarakham 44150, Thailand; 5 Department of Science and Technology, Faculty of Liberal Arts and Science, Roi-Et Rajabhat University, Sela Phum District, Roi-Et 45120, Thailand; onanong.p@reru.ac.th

**Keywords:** watermeal, starch digestion, protein digestion, glycemic index, bioactive compounds

## Abstract

This study examined the impact of incorporating watermeal (*Wolffia globosa*) on the physicochemical characteristics, antioxidant activity, and starch and protein digestibility of rice noodles. The addition of watermeal powder (1, 3, 5%) significantly enhanced the nutritional and functional attributes of the noodles. The formulation with 5% watermeal (WF5) demonstrated a twofold increase in protein content compared to the control, along with a marked increase in the chlorophyll content as the watermeal concentration increased (*p* < 0.05). Moreover, fortifying the noodles with watermeal enhanced their bioactive compound content and antioxidant activity in all fortified noodles. Starch digestibility analyses revealed an increase in the resistant starch and slowly digestible starch, along with a reduction in the rapidly digestible starch and the estimated glycemic index. Protein digestibility in the WF5 sample improved by 22% compared to the control. These findings emphasize the capability of watermeal as a sustainable, plant-based ingredient for developing nutrient-rich noodle products with enhanced health benefits.

## 1. Introduction

*Wolffia globosa* (Roxb.) Hartog and Plas, also known as watermeal or duckweed, is referred to in Thai as phum, khai-phum, or khai-nam. Watermeal belongs to the Lemnaceae family and is characterized by its unique plant structures and ecological niches [1]. In recent years, this aquatic plant has attracted significant attention due to its exceptional nutritional profile, particularly as a sustainable protein source [2], with genotoxicity and repeated-dose toxicity evaluations also conducted [3]. Recently, various plant-based proteins have gained commercial availability and experienced rapid growth in the food industry [4]. Watermeal is rich in essential amino acids, vitamins, minerals, and bioactive compounds, making it a valuable ingredient for the development of plant-based foods. The growing demand for sustainable and nutritionally balanced alternatives to conventional food products has driven the exploration of watermeal in innovative food formulations, including plant-based noodles. It is valued as an affordable and accessible protein source, particularly in areas where staple foods, such as noodles and rice, are predominantly carbohydrate-rich. Given this historical use and nutritional value, *Wolffia* species are increasingly seen as promising ingredients for enhancing protein content in starch-based diets [5]. Particularly, watermeal has gained attention as a sustainable and nutrient-dense ingredient for food applications, containing 38% carbohydrates, 24% protein, 20% ash, and 3% fat [6].

Noodles are a widely consumed staple food [7], valued for their versatility, convenience, and affordability, which make them accessible to a wide range of consumers [8]. As a dietary staple in many cultures, noodles are made from various ingredients, including wheat, rice, and other starches, primarily serving as a vital source of carbohydrates [9]. However, despite their widespread popularity and adaptability in various cuisines, traditional noodles often lack essential nutrients, such as protein, dietary fiber, vitamins, and bioactive compounds [10]. This nutritional gap limits their role in providing a balanced diet, particularly for health-conscious consumers seeking more functional and nutrient-dense food options. Therefore, incorporating watermeal into noodle formulations can enhance their nutritional value, offering a solution for foods with a low nutrient profile.

There is also evidence suggesting that incorporating watermeal can enhance the nutritional profile of snack products [11]. Noodles hold significant cultural and dietary importance worldwide, and their nutritional composition largely depends on the type of flour and ingredients used [9]. As consumer demand grows for healthier, more sustainable, and nutrient-dense alternatives to traditional rice-based noodles, plant-based noodles have gained considerable attention [12]. Their rising popularity reflects an increasing consumer preference for environmentally friendly and healthy food options. The incorporation of watermeal into noodle formulations is particularly promising due to the plant’s protein content, digestibility, and health-promoting bioactive compounds, such as antioxidants and dietary fibers [13].

Therefore, this research aimed to develop a watermeal-enriched noodle product, focusing on a comprehensive evaluation of its chemical and physical properties, biological activity, and starch and protein digestibility through simulated *in vitro* digestion. Such studies are essential to determine the potential of plant-based noodle alternatives that can meet consumer needs for nutrition, functionality, and sustainability.

## 2. Materials and Methods

### 2.1. Materials

Watermeal (*W. globosa*) was harvested from a farm in Nakhon Ratchasima Province, Thailand. After washing, the sample was dried in a hot air oven at 60 °C to a moisture content of approximately 14% (dry basis) and then finely milled to a particle size of 50 mesh for further use. Tapioca starch and rice flour were obtained from a local supermarket in Maha Sarakham province, Thailand.

### 2.2. Preparation of Noodle Samples

The samples were prepared following the method outlined by Thongkaew and Singthong [14], with slight modifications. Specifically, as detailed in Table 1, all ingredients were thoroughly blended for 2 min before being steamed at 90 °C for 10 min. After steaming, the mixture was sliced into uniform strips and dried in a hot air oven (Intro Enterprise Co., Ltd., Bangkok. Thailand) at 60 °C for 5 h. The dried samples were then stored for further analysis.

### 2.3. Analysis of Physical Properties

#### 2.3.1. Color

The noodle color was evaluated using a CR-400/CR-410 colorimeter (Chroma Meter; Konica Minolta Sensing Inc., Osaka, Japan), with modifications based on the described method [15]. The results were expressed using the CIELab color space system of the International Commission on Illumination (Commission Internationale de L’Eclairage). Each sample was measured five times. Approximately 5 g of the noodles was ground using a mortar and pestle and placed into a small, sealed plastic bag for the measurement.

#### 2.3.2. Water Activity

The water activity (a_w_) was measured using a Novasina RS 200 water activity meter (Axair Ltd., Pfaffikon, Switzerland).

#### 2.3.3. Textural Properties

The textural profile of cooked noodle samples was measured using a TA-XT plus texture analyzer (Stable micro systems, Surrey, UK) equipped with a P/35 probe. The variables for the compression mode were set as follows: the pre-test speed was 1.0 mm/s, the test speed was 0.8 mm/s, and the post-test speed was 0.8 mm/s. The strain was set at 70%, and the trigger type was set to auto with a 5 g threshold [16].

To evaluate the tensile properties, a single noodle strand was secured around the parallel rollers of the Texture Analyzer using the A/SPR probe to ensure no slippage. The testing conditions were set as follows: the pre-test speed was 1.0 mm/s, the test speed was 3.0 mm/s, the post-test speed was 10.0 mm/s, the distance was 100 mm, and the impulse force was 5 g. The final textural property values for each sample were calculated as the average of five measurements.

### 2.4. Determination of Cooking Parameters

#### 2.4.1. Cooking Time

The cooking time of the noodle samples was determined using a method adapted from AACC [17]. A 10 g noodle sample was boiled in 1 L of distilled water, with the cooking time being recorded. The noodles were considered fully cooked when their center appeared clear, indicating the absence of cloudiness.

#### 2.4.2. Cooking Loss

The method for assessing the cooking loss of rice noodle samples was adapted from AACC [17] with slight modifications. Specifically, 15.0 g of rice noodle samples were added to 150 mL of boiling distilled water and cooked for 20 min. After cooking, the noodles were removed and rinsed with distilled water using a Büchner funnel. The combined cooking and rinse water was collected in a pre-weighed beaker. To determine the cooking loss, the collected water was evaporated to dryness in an oven at 105 °C. The cooking loss was then calculated and expressed as a percentage of the residue.

(1) $$\mathrm{Cooking}\;\mathrm{loss}\;(\%)=\frac{Weight\;of\;dried\;residue\;in\;cooking\;water\;and\;rinse\;water}{Weight\;of\;fresh\;noodles}\times 100$$

#### 2.4.3. Cooking Yield

Approximately 10 g of noodles were cooked in a beaker for the optimal cooking time, rinsed with distilled water, and drained for 15 min before weighing. The cooking yield was then evaluated using the following equation:

(2) $$\mathrm{Cooking}\;\mathrm{yield}\;(\%)=\frac{Cooked\;noodles\;weight}{Fresh\;noodles\;weight}\times 100$$

#### 2.4.4. Water Absorption Capacity (WAC)

To evaluate water absorption during the cooking process, 15 g of noodle samples were immersed in 150 mL of boiling distilled water and cooked for 20 min. After cooking, the noodles were drained and weighed. Water absorption was determined by calculating the weight difference between the cooked and uncooked samples and expressing it as a percentage [18].

(3) $$\mathrm{WAC}\;(\%)=\frac{Weight\;of\;cooked\;noodle-Weight\;of\;uncooked\;noodle}{Weight\;of\;uncooked\;noodle}\times 100$$

### 2.5. Scanning Electron Microscopy

According to the method detailed by Tang et al. [19], scanning electron microscopy (SEM) was used to examine the microstructure of both uncooked and cooked samples (S-4800Ⅱ, Hitachi, Tokyo, Japan). The analysis was conducted at 2000× magnification and an accelerating voltage of 10 kV.

### 2.6. Analysis of Proximate Composition

The quantification of moisture (925.10), ash (900.02), protein using Kjeldahl methods (920.176), fat (920.177), and fiber (985.29) was performed according to the AOAC methods [20]. The carbohydrate content was calculated using the equation below:

(4) $$\mathrm{Carbohydrate}\;\mathrm{quantity}\;(\%)=100-\left(\%ash+\%protein+\%fat+\%fiber\right)$$

### 2.7. Determination of Chlorophyll Content

This method was adapted from Zou et al. [21]. The chlorophyll in watermeal and watermeal-enriched noodles was extracted using 80% acetone. The samples (0.1 g dry weight) were mixed with 80% acetone (*v*/*v*) and left in the dark until the sample turned white. Absorbance was measured at 645 and 663 nm, and the chlorophyll content was calculated using the following formula [22].

(5) $$\mathrm{Chlorophyll}\;\mathrm{content}\;(\mathrm{mg}/100\;g)=\left(\left(8.02\times OD663+20.21\times OD645\right)\times \frac{V}{S\times 1000}\right)*100$$

where V is the liquid volume and S is the weight of the sample.

### 2.8. Antioxidant Activity Analysis

#### 2.8.1. Extraction Procedure for Phenolic-Antioxidant Compounds

The extraction method was adapted from Jelled et al. [23]. Briefly, 5 g of cooked noodle samples was thoroughly mixed with 30 mL of 99.99% ethanol and agitated on an orbital shaker at 150 rpm for 12 h at room temperature. The mixture was then filtered using Whatman filter paper. The obtained extract was used for the analysis of phenolic compounds, flavonoids, and antioxidant activity.

#### 2.8.2. Total Phenolic Content

The total phenolic content (TPC) was determined using the Folin–Ciocalteu method, as described by Ratseewo et al. [24]. The Folin–Ciocalteu reagent (diluted to a 1:10 ratio with distilled water) was prepared, and 500 µL of this solution was mixed with 200 µL of each extract. The mixture was incubated at 25 °C for 5 min, followed by the addition of 2250 µL of a 7.5% sodium carbonate solution. The reaction mixture was left at room temperature for 90 min. Absorbance was measured at 725 nm using a UV spectrophotometer (DR 2700, HACH, Loveland, CO, USA). A calibration curve was generated using gallic acid solutions (1–100 mg/L), and the TPC was determined and reported as mg of gallic acid equivalents per 100 g of sample on a dry weight basis (mg GAE/100 g db).

#### 2.8.3. Total Flavonoid Content

The total flavonoid content (TFC) was quantified using the colorimetric method outlined by Siriamornpun and Kaewseejan [25]. An aliquot of 0.5 mL of the extracted sample was mixed with 2.25 mL of distilled water and 0.15 mL of 5% (*w*/*v*) NaNO_2_ solution. After 6 min, 300 µL of 10% (*w*/*v*) AlCl_3_ solution was added, followed by 5 min incubation. Subsequently, 100 µL of NaOH solution at a concentration of 1 mol/L was introduced. The absorbance of the final solution was measured at 510 nm using a UV-1700 spectrophotometer (Shimadzu, Tokyo, Japan). The TFC was expressed as mg of quercetin equivalents per 100 g of the sample (mg QE/100 g db).

#### 2.8.4. 1,1-Diphenyl-2-Picrylhydrazyl (DPPH) Radical Scavenging Activity

The DPPH free radical scavenging capacity of extracted samples was determined following Siriamornpun et al. [26]. The extract (500 μL) was mixed with 4.5 mL of freshly prepared DPPH solution (0.1 mmol/L methanol). The prepared mixture was vortexed thoroughly and kept in the dark for 30 min at ambient temperature. Absorbance was subsequently measured at 517 nm. The results were calculated using a standard curve of vitamin C and expressed as mg of vitamin C per 100 g of sample (mg vitamin C/100 g db).

#### 2.8.5. Ferric Reducing Antioxidant Power (FRAP)

The extract’s reducing power was measured through the FRAP assay, as described by Siriamornpun et al. [26]. The freshly prepared FRAP reagent was made by mixing 100 mL of acetate buffer (300 mmol/L, pH 3.6), 10 mL of 2,4,6-Tris (2-pyridyl)-s-triazine (TPTZ) solution dissolved in 40 mmol/L HCl, and 10 mL of 20 mmol/L FeCl_3_ in a 10:1:1 ratio, followed by the addition of 12 mL of distilled water at 37 °C. In brief, 60 μL of the sample solution and 180 μL of deionized water were mixed with 1.8 mL of the FRAP reagent. The mixture was vortexed thoroughly. After incubation at 37 °C for 4 min in a water bath, the absorbance was measured at 593 nm against a blank. The FRAP values were expressed as mg of FeSO_4_ per 100 g (mg FeSO_4_/100 g db).

### 2.9. HPLC Analysis of Phenolic Acid and Flavonoid Compositions

Phenolic acids and flavonoids were extracted using the method described by Chumroenphat et al. [27] and Kubola et al. [28]. For HPLC analysis, a 20 μL aliquot of the sample solution was fractionated using the HPLC system (Series 20, Shimadzu, Kyoto, Japan) equipped with a diode array detector and a 250 mm × 4.6 mm, 5 μm, Inertsil C18 analytical column. The mobile phase consisted of purified water containing 1% (*v*/*v*) acetic acid (solvent A) and acetonitrile (solvent B) at a flow rate of 0.8 mL/min. The column temperature was set at 38 °C. Phenolic acid and flavonoid compounds in the samples were identified using external standards for calibration.

### 2.10. Analysis of In Vitro Protein Digestibility

The simulated gastrointestinal samples were digested following the INFOGEST protocol [29]. Simulated salivary fluid (SSF), gastric fluid (SGF), and intestinal fluid (SIF) were prepared for the digestion process. Initially, four milliliters of purified water were added to one gram of rice noodles. For the oral digestion phase, SSF was added at a 1:1 (*w*/*w*) ratio to achieve a paste-like appearance, followed by the addition of amylase from saliva (75 U/mL). The bolus was continuously stirred at pH 7.0 and 37 °C for two minutes during incubation. To simulate the gastric digestion stage, the mixture was diluted 1:1 (*v*/*v*) with SGF, pepsin (2000 U/mL) was added, and the digestion proceeded at pH 3.0 and 37 °C for 2 h. To simulate the intestinal digestion stage, the gastric digest was further diluted 1:1 (*v*/*v*) with SIF, supplemented with bile salts (10 mmol/L in the final mixture) and pancreatin (100 U/mL trypsin activity in the final mixture), and the simulated intestinal phases were incubated at pH 7.0 and 37 °C for 2 h. Following digestion, the samples were centrifuged, and the supernatant was stored at −20 °C until further analysis. Protein digestibility (%) was calculated by determining the ratio of the protein content in the digests (measured using the Bradford assay) to the protein content of the original sample.

### 2.11. Measurement of Total Sulfhydryl Content

The total sulfhydryl (TSH) content was determined using a modified method of Ellman [30]. Briefly, 0.1 g of sample was dispersed in 8 mL of 0.1 mol/L phosphate buffer (pH 7.2, urea (8 mol/L), sodium dodecyl sulfate (SDS) (2%), and ethylene diamine tetraacetic acid (EDTA) (10 mmol/L). Subsequently, 4 mL of the mixture was added into 0.4 mL of a 0.1% 5,5′-dithiobis (2-nitrobenzoic acid) (DTNB) solutiminon, followed by a 15 min incubation in a dark room. The absorbance was measured at 412 nm using a UV spectrophotometer (DR 2700, HACH, Loveland, CO, USA). The TSH content, expressed in micromoles per gram of protein (µmol/g), was determined using an extinction coefficient of 13,600 M^−1^·cm^−1^.

### 2.12. Analysis of In Vitro Starch Digestibility

*In vitro* starch digestion was determined using the method described by Englyst et al. [31] with slight modifications. Briefly, 200 mg of the sample was thoroughly mixed with 15 mL of sodium acetate buffer (200 mmol/L, pH 5.2). Hydrolysis was initiated by adding 5 mL of alpha-amylase (290 U/mL) and 5 mL of amyloglucosidase (15 U/mL), followed by incubation at 160 rpm and 37 °C. Aliquots (1 mL) of the hydrolysate were collected at 0, 20, 30, 60, 90, 120, 150, and 180 min and immediately mixed with 4 mL of 66% (*v*/*v*) ethanol. The mixture was then centrifuged at 6000 rpm for 10 min (Allegra X-30R, Beckman Coulter, Brea, CA, USA), and glucose levels in the supernatant were determined using the 3,5-Dinitrosalicylic acid (DNS) method. The estimated glycemic index (eGI) was calculated using the following formula:

eGI = 8.198 + 0.862 × HI(6)

where the hydrolysis index (HI) is defined as the ratio of the area under the sample’s hydrolysis curve to the area under the white bread hydrolysis curve.

### 2.13. Fourier Transform Infrared (FTIR) Measurement

An FTIR spectrometer (670-IR+610-IR, Varian, Palo Alto, CA, USA) was used to record the FTIR spectra of the cooked noodle samples. In brief, 1 mg of noodle sample was mixed with KBr (1:100, *w*/*w*), and the mixture was ground thoroughly and pressed for 5 min to form a KBr pellet. The FTIR absorption spectra were recorded from 400 to 4000 cm^−1^ at a resolution of 4 cm^−1^, with 32 scans.

### 2.14. Evaluation of Sensory Attributes

The sensory acceptability of the noodles was evaluated based on appearance, color, aroma, taste, texture, and overall liking using a 9-point hedonic scale (1 = dislike extremely to 9 = like extremely) [11] with 30 panelists (ages 18–50 years old, no history of allergy to ingredients used). Each panelist received a 15 g sample served in a bowl, labeled with a three-digit random code to minimize bias. Drinking water was provided for palate cleansing between tastings. This experiment was conducted in accordance with ethics approval number 195-822/2568.

### 2.15. Statistical Analysis

The results represent the mean ± SD obtained from at least three independent trials. The data were subjected to one-way ANOVA followed by the Duncan’s multiple range test. Significant differences were determined at *p* < 0.05 by IBM SPSS Statistical Software version 17.0.

## 3. Results and Discussion

### 3.1. Physical Properties and Chlorophyll Content

**Figure 1:**
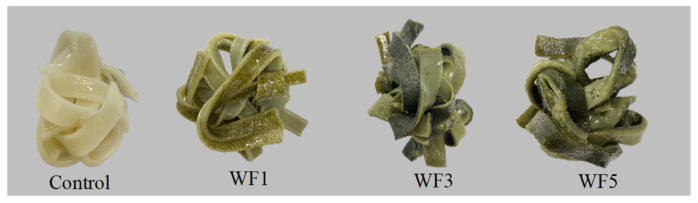
Appearances of cooked watermeal noodles compared to control sample. Control = no watermeal, WF1 = 1% watermeal, WF3 = 3% watermeal, WF5 = 5% watermeal.

All noodle samples in Table 2 exhibited a_w_ values ranging from 0.47 to 0.48, effectively inhibiting microbial growth, as it falls below the threshold required for microbial activity. The incorporation of watermeal influenced the noodle color, with L*, a*, and b* values ranging from 43.33 to 80.65, −4.24 to −0.19, and 7.76 to 18.73, respectively. As the percentage of watermeal increased, lightness (L*) significantly decreased, while greenness (a*) and yellowness (b*) significantly increased. A higher watermeal proportion resulted in a more intense green color, as shown in Figure 1. The addition of watermeal increased the chlorophyll content, leading to a significant color change compared to the control sample, consistent with findings by On-Nom et al. [11]. Table 3 presents the textural properties of the noodles. The hardness of noodles supplemented with 1% (WF1), 3% (WF3), and 5% (WF5) watermeal was significantly higher than that of the control. As a key indicator of overall texture quality, hardness plays a crucial role in determining the firmness and structural integrity of the noodles [32]. Cohesiveness, defined as the extent to which the bolus retains its structure after mastication [33], decreased with higher levels of watermeal, indicating that these noodles disintegrated more easily. Adhesiveness exhibited trends similar to those observed for cohesiveness. The interaction between components such as protein in rice flour with starch molecules typically forms a stable structure [34]. However, excessive watermeal addition may disrupt this interaction, leading to increased hardness and reduced cohesiveness and adhesiveness of the noodles.

### 3.2. Cooking Parameters

Table 4 summarizes the cooking times, which ranged from 9.52 to 11.22 min, increasing with higher levels of watermeal. The control group had the shortest cooking time (9.25 min), while WF5 required the longest cooking time (11.22 min). This suggested that the higher protein content from watermeal addition slowed the cooking process, aligning with the findings reported by Yao et al. [35]. The cooking loss of noodles increased with watermeal addition. WF3 and WF5 exhibited cooking losses of 2.07% and 2.35%, respectively, which were significantly higher than that of the control sample (0.95%), except for WF1, which did not show a significant difference compared to the control. These results indicated that excessive watermeal addition promoted noodle disintegration during cooking, aligning with findings by Sun et al. [36]. This effect likely results from watermeal interfering with amylose network formation, as suggested by Geng et al. [37], leading to a weaker noodle structure. Water absorption, defined as the ability of the noodles to retain water, is influenced by components such as starch, fiber, and protein composition, as well as the strength of the protein or starch network [21]. The water absorption capacity (WAC) of WF5 differed significantly from the control, while WF1 and WF3 showed no significant difference between them. However, both WF1 and WF3 exhibited significant differences compared to the control. This suggests that higher watermeal levels may disrupt the structural network responsible for water retention.

### 3.3. Microstructure

**Figure 2:**
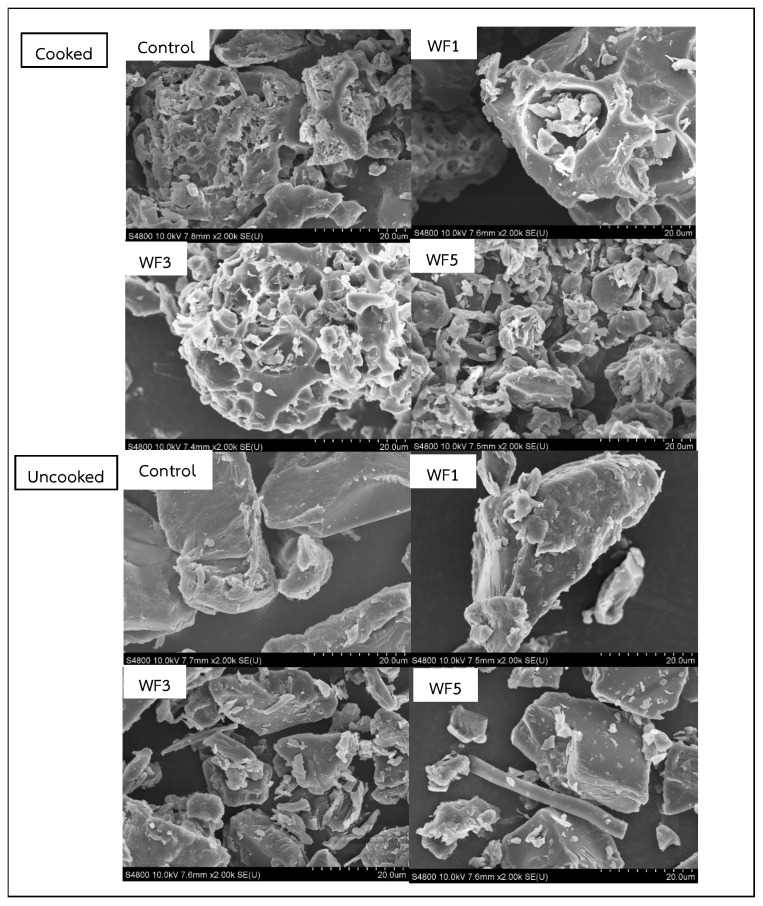
Comparison of cooked and uncooked composite watermeal noodles to control sample through SEM imaging. Control = no watermeal, WF1 = 1% watermeal, WF3 = 3% watermeal, WF5 = 5% watermeal.

The microstructural changes in uncooked and cooked rice noodles with different watermeal formulations are shown in Figure 2. In the control sample, most of the starch granules swelled and aggregated into large starch lumps. The outer layer of the cooked noodles appeared quite porous, as observed in the WF3 sample, which aligns with the findings reported by Yao et al. [35]. In the WF5 sample, a significant number of intact starch granules were observed compared to the protein network structure. The control sample exhibited a less continuous and dense network, whereas WF5 displayed a continuous protein network that more tightly surrounded the starch granules, providing structural integrity [38,39], thereby inhibiting enzyme access to the starch [40]. Additionally, a relatively dense microstructure was observed in all uncooked noodle samples, indicating the presence of numerous starch granules embedded within the protein network. This embedding enhances the interaction between the protein and starch in raw noodles, preventing the starch granules from swelling during cooking and reducing the starch hydrolysis process later. Changes in the microstructure of both cooked and raw noodles also demonstrated this improvement, as similarly reported by Tian et al. [41].

### 3.4. Proximate Composition

The proximate composition of noodles with watermeal and control noodles is presented in Table 5. WF5 had the highest protein (8.39%), lipid (4.75%)—containing both saturated and unsaturated fatty acids, as reported by Boonarsa [1]—ash (1.95%), and fiber (2.74%) contents compared to other samples (control, WF1, and WF3). WF5 exhibited nearly double the protein content compared to the control, although carbohydrate content was slightly lower. This finding aligns with On-Nom et al. [11], who reported that snacks supplemented with *Wolffia* contained up to 12.38% protein. Similarly, dried duckweed powder has demonstrated a relatively high protein content of 24%, highlighting its potential as an alternative protein source [6].

### 3.5. Antioxidant Properties

**Figure 3:**
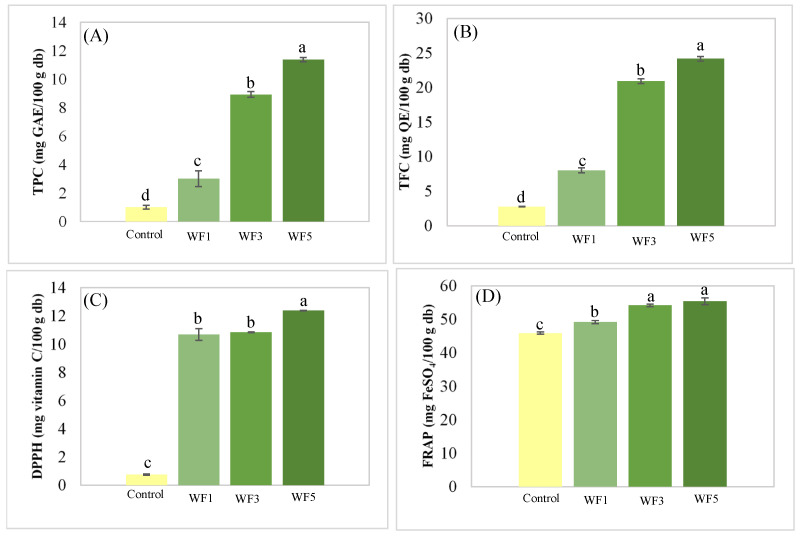
Antioxidant properties of noodles: (**A**) total phenolic content (TPC); (**B**) total flavonoid content (TFC); (**C**) DPPH radical scavenging activity; (**D**) ferric reducing antioxidant power (FRAP). Column and error bar represent mean and standard deviation (*n* = 3), respectively. Values with different letters are considered as significantly different (*p* < 0.05). Control = no watermeal, WF1 = 1% watermeal, WF3 = 3% watermeal, WF5 = 5% watermeal.

The phytochemical contents, including TPC and TFC, along with antioxidant properties such as DPPH radical scavenging and FRAP activities, were analyzed to evaluate the health benefits of the watermeal-fortified noodles. Figure 3 shows that WF5 had the highest TPC and TFC levels, with values of 11.37 mg GAE/100 g db and 24.16 mg QE/100 g db, respectively. Antioxidant activity, closely correlated with phytochemical levels, was also the highest in WF5 among all tested samples. These findings indicated that the addition of watermeal significantly enhanced the phytochemical content and antioxidant properties of the noodles. Similar trends have been observed in studies on snacks fortified with watermeal powder (WP), where the addition of WP increased both phytochemical content and antioxidant activities, reinforcing the potential of watermeal as a functional food ingredient. These results align with previous reports demonstrating the ability of watermeal to enhance the nutritional and bioactive characteristics of food products [11,35]. Further studies, such as those by Dhamaratana et al. [6] and Geng et al. [36], also emphasize the potential of aquatic plants like watermeal in delivering health benefits through their rich phytochemical profiles and antioxidant activities.

### 3.6. Compositions of Phenolic Acids and Flavonoids

The incorporation of watermeal powder into rice noodles significantly influenced the composition of phenolic acids and flavonoids. As shown in Table 6, the total phenolic acid content increased with higher levels of watermeal supplementation. Interestingly, gallic acid and protocatechuic acid were detected only in WF3 and WF5 samples, with the highest concentrations observed in WF5 (100.21 µg/g and 3.28 µg/g, respectively). The absence of detectable gallic acid in WF1 may be due to its low concentration, which falls below the detection limit of HPLC [42]. These results align with prior studies demonstrating that watermeal, similar to duckweed, is a rich source of phenolic compounds, contributing to its functional food potential [43]. Similarly, the p-coumaric acid, ferulic acid, and cinnamic acid levels increased significantly (*p* < 0.05) in watermeal-fortified samples compared to the control. Flavonoid content also exhibited a marked increase in watermeal-fortified noodles. Rutin was absent in the control but was detected in all watermeal samples, with the highest concentration in WF5 (33.76 µg/g). Quercetin levels increased slightly across samples, while apigenin and karmferal concentrations significantly rose with higher watermeal supplementation, reaching peak values of 140.04 µg/g and 33.04 µg/g in WF5, respectively. These findings suggest that fortifying rice noodles with *W. globosa* enhances their bioactive compound content, particularly phenolic acids and flavonoids, which are known for their potential health benefits. The results indicate that *W. globosa* could serve as a valuable ingredient for enriching rice noodles with bioactive compounds, offering potential functional food applications. The increasing concentrations of gallic acid and apigenin with higher watermeal levels confirm the effectiveness of fortifying noodles with *W. globosa*. This enrichment aligns with findings by Yao et al. [35], who demonstrated the phytochemical enhancement of plant-based food products through functional ingredient addition. Furthermore, the phytochemical profile of *W. globosa* supports its reported antioxidant and anti-inflammatory activities, as discussed by Dhamaratana et al. [6] and Geng et al. [36]. These findings underscore the potential of watermeal as a sustainable and nutrient-rich ingredient for improving the bioactive properties of food products, contributing to both health and functional food innovation.

### 3.7. In Vitro Protein Digestibility

Protein digestibility is a crucial measure of protein quality, reflecting its availability for human nutrition. *W. globosa* (watermeal) is recognized as a highly valuable protein source, containing over 45% protein by weight and all nine essential amino acids required for human health [44]. Simulated digestion studies closely monitored protein digestibility across various formulations, as summarized in Table 7. The results indicated that noodles fortified with watermeal exhibited higher protein digestibility compared to the control group. This effect was particularly pronounced in WF5, where the higher protein digestibility (81.51%) may be attributed to the interaction between watermeal proteins and the noodle matrix, potentially facilitating enzyme access and protein hydrolysis. Similar trends have been observed in previous studies [6,45], where the food matrix structure influenced digestion efficiency. These findings align with prior research, demonstrating that plant-based proteins, such as those from legumes, lentils, and aquatic plants, contribute to improved protein digestibility and nutritional quality [46,47]. This characteristic highlights watermeal as a promising ingredient for enhancing protein bioavailability in functional food products.

### 3.8. Total Sulfhydryl Content

Sulfhydryl content is a key protein conformational element affecting functionality. Processing methods such as heating and high pressure can degrade sulfhydryl groups, impacting protein properties. Table 7 shows the TSH content in rice noodles, with fortified noodles (2.25–4.44 μmol/g) having higher values than the control (2.24 μmol/g). WF5 had the highest TSH, followed by WF3, with TSH increasing in proportion to the watermeal concentrations. The increase in TSH with watermeal fortification may be linked to the higher protein content in watermeal powder, which introduces cysteine and other sulfur-containing amino acids [1]. The sulfhydryl groups (–SH) in cysteine form disulfide bonds, contributing to protein stability. During noodle processing, heat and mechanical stress expose sulfhydryl groups, increasing disulfide content, as seen in wheat noodles [48].

### 3.9. In Vitro Starch Digestibility

The changes in starch digestibility of uncooked and cooked noodles with varying levels of watermeal are presented in Table 8. Compared to the control samples, the noodles with watermeal showed significantly increased contents of resistant starch (RS) and slowly digestible starch (SDS), whereas the levels of rapidly digestible starch (RDS) and estimated glycemic index (eGI) were significantly lower. The incorporation of watermeal contributed to a marked rise in RS, indicating enhanced resistance to digestion and highlighting the potential of watermeal as a functional ingredient. While the addition of watermeal tended to increase SDS and RS levels, the RDS content displayed a significant reduction with increasing watermeal levels. This pattern is consistent with findings from other studies on plant-based protein-enriched noodles, such as those supplemented with rice bran [32], where protein–starch interactions altered digestibility properties. The results suggested that watermeal acted as a functional ingredient, modifying starch digestion behavior by increasing the RS and SDS content and lowering the RDS content and eGI. These changes make watermeal-fortified noodles an excellent dietary option for individuals managing blood glucose levels or seeking low-glycemic foods. Similar trends in RS and RDS were observed in protein-enriched food systems, such as pea protein–starch matrices [49] and lentil protein-enriched noodles [50]. Future research should explore the detailed mechanisms underlying protein–starch interactions during processing and digestion, with a particular focus on the unique contributions of watermeal proteins.

### 3.10. Fourier-Transformation Infrared Spectra

**Figure 4:**
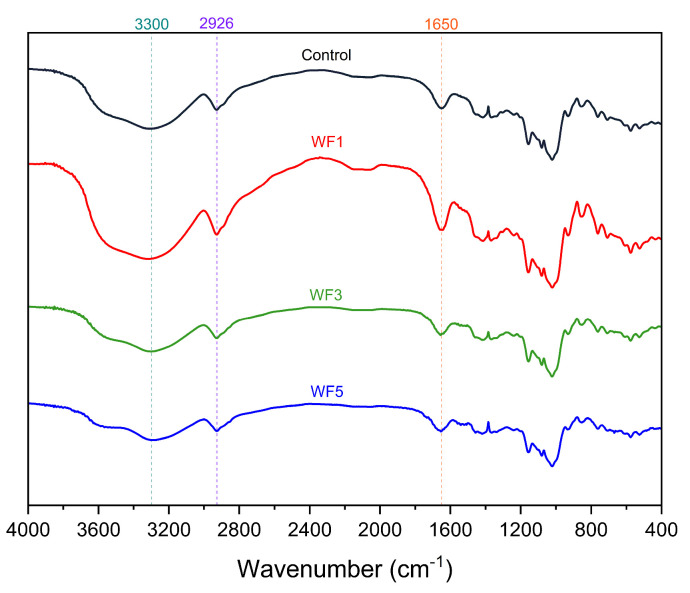
Fourier−transform infrared spectra of cooked noodle samples. Control = no watermeal, WF1 = 1% watermeal, WF3 = 3% watermeal, WF5 = 5% watermeal.

Figure 4 illustrates the FTIR spectra of various cooked noodle formulations, highlighting the molecular organization and interactions among their components. All formulations exhibited FTIR spectra within the range of 400–4000 cm^−1^, showing distinct absorption bands that correspond to different functional groups present in the noodle matrix. A strong absorption peak at approximately 1650 cm^−1^ corresponds to amide I, which is attributed to the C=O stretching vibrations of the acetylated amino group [51,52]. The broad absorption band observed around 3300 cm^−1^ is indicative of O–H stretching vibrations, which suggests the presence of hydroxyl groups, likely originating from water, starch, and other hydrophilic components in the noodle formulations. This observation aligns with findings reported by Yang et al. [53] and Daeialiakbar et al. [54]. Furthermore, additional absorption peaks around 2926 cm^−1^ can be attributed to C–H stretching vibrations, which are typically associated with the aliphatic hydrocarbon chains of lipids, proteins, and carbohydrates. The spectrum also reveals characteristic bands [55], which may correspond to specific molecular interactions within the noodle structure. These spectral features provide insights into the chemical composition and potential modifications that occur during the cooking process.

### 3.11. The Sensory Evaluation

The sensory evaluation assessed the acceptability of watermeal-fortified noodles compared to the control sample using a 9-point hedonic scale (Table 9). The control sample received the highest scores for appearance, color, and taste. The texture liking scores of WF1 and the control sample showed no significant difference, as well as the overall liking scores. Among the watermeal-fortified noodles, WF1 had the highest overall acceptability score (7.30), indicating better acceptance compared to WF3 and WF5. However, its overall acceptability was not significantly different from the control sample, which had a score of approximately 6, consistent with previous findings reported by On-Nom et al. [11].

## 4. Conclusions

Our present study has shown that the incorporation of watermeal (*W. globosa*) into rice noodles significantly improved their nutritional and functional properties, highlighting its potential as a sustainable plant-based ingredient. The addition of watermeal enhanced the protein content, chlorophyll levels, and antioxidant activity while enriching the noodles with bioactive compounds. Additionally, the fortified noodles exhibited improved starch digestibility, characterized by increased RS and SDS, reduced RDS, and a lower eGI. Protein digestibility also improved significantly with an increase in the watermeal content compared to the control. These findings suggest that watermeal can be effectively exploited in the development of nutrient-dense, functional foods. Future applications may include its use in gluten-free or high-protein noodles, tailored for health-conscious consumers or individuals with specific dietary needs, such as diabetics or those seeking plant-based protein alternatives. Additionally, the scalability of watermeal cultivation could support sustainable food production, aligning with global goals for food security and environmental sustainability.

## Figures and Tables

**Table 1:** Ingredient compositions of watermeal noodle formulations.

Ingredient (%)	Control	WF1	WF3	WF5
Rice Flour	16.70	16.70	16.70	16.70
Tapioca Starch	20.80	19.80	17.80	15.80
Water	62.50	62.50	62.50	62.50
Watermeal	0.00	1.00	3.00	5.00

Note: Watermeal powder replaces tapioca starch; control = no watermeal; WF1 = 1% watermeal; WF3 = 3% watermeal; WF5 = 5% watermeal.

**Table 2:** Physical properties and chlorophyll content of watermeal noodles in comparison to control sample.

Sample	L*	a*	b*	a_w_	Chlorophyll (mg/100 g)
Control	80.65 ± 1.41 a	−0.19 ± 0.14 a	7.76 ± 0. 41 d	0.48 ± 0.00 a	0.24 ± 0.03 c
WF1	61.94 ± 2.17 b	−1.88 ± 0.08 b	12.35 ± 1.26 c	0.48 ± 0.00 a	11.18 ± 0.06 b
WF3	47.55 ± 1.79 c	−3.24 ± 0.38 c	17.10 ± 0.39 b	0.48 ± 0.00 a	31.00 ± 0.33 a
WF5	43.33 ± 1.91 d	−4.24 ± 0.15 d	18.73 ± 0.41 a	0.47 ± 0.00 b	32.11 ± 0.20 a

Results are expressed as means ± standard deviation (*n* ≥ 3). Different letters within the same column indicate significant differences (*p* < 0.05). Control = no watermeal, WF1 = 1% watermeal, WF3 = 3% watermeal, WF5 = 5% watermeal.

**Table 3:** Textural properties of cooked watermeal noodles in comparison to control sample.

Sample	Tensile Strength (N)	Hardness (N)	Adhesiveness (N·s)	Springiness (%) ^ns^	Cohesiveness
Control	0.78 ± 0.00 a	56.97 ± 3.83 b	2.59 ± 0.28 a	99.95 ± 0.02	0.82 ± 0.05 a
WF1	0.41 ± 0.00 b	74.67 ± 7.67 a	2.56 ± 0.13 a	99.98 ± 0.02	0.77 ± 0.01 ab
WF3	0.33 ± 0.00 c	78.01 ± 4.83 a	2.30 ± 0.17 b	99.98 ± 0.01	0.68 ± 0.08 b
WF5	0.27 ± 0.00 d	84.12 ± 9.31 a	1.70 ± 0.20 c	99.97 ± 0.01	0.66 ± 0.07 b

Results are expressed as means ± standard deviation (*n* = 5). Different letters within the same column indicate significant differences (*p* < 0.05); ns = not significantly different (*p* > 0.05). Control = no watermeal, WF1 = 1% watermeal, WF3 = 3% watermeal, WF5 = 5% watermeal.

**Table 4:** Comparison of cooking properties of watermeal noodles with control sample.

Cooking Parameter	Control	WF1	WF3	WF5
Cooking time (min)	9.52 ± 0.12 c	10.03 ± 0.35 b	10.56 ± 0.13 b	11.22 ± 0.02 a
Cooking loss (%)	0.95 ± 0.02 b	1.69 ± 0.10 ab	2.07 ± 0.05 a	2.35 ± 0.16 a
Cooking yield (%)	289.45 ± 3.45 a	281.84 ± 2.33 b	276.34 ± 2.36 b	261.48 ± 0.95 c
Water absorption capacity (%)	189.45 ± 3.45 a	181.84 ± 2.33 b	176.34 ± 2.36 b	161.48 ± 0.95 c

Results are expressed as means ± standard deviation (*n* = 3). Different letters within the same row indicate significant differences (*p* < 0.05). Control = no watermeal, WF1 = 1% watermeal, WF3 = 3% watermeal, WF5 = 5% watermeal.

**Table 5:** Comparison of proximate composition of watermeal noodles with control sample (dry weight basis).

	Control	WF1	WF3	WF5
Moisture (%) ^ns^	7.78 ± 0.01	7.81 ± 0.32	7.34 ± 0.28	7.72 ± 0.56
Ash (%)	1.64 ± 0.05 d	1.76 ± 0.01 c	1.84 ± 0.03 b	1.95 ± 0.01 a
Protein (%)	4.29 ± 0.30 d	4.87 ± 0.05 c	6.18 ± 0.49 b	8.39 ± 0.54 a
Fat (%)	2.06 ± 0.02 c	2.35 ± 0.04 c	3.76 ± 0.03 b	4.75 ± 0.01 a
Fiber (%)	1.42 ± 0.01 d	1.65 ± 0.00 c	2.16 ± 0.02 b	2.74 ± 0.03 a
Carbohydrate (%)	93.06 ± 0.67 a	89.5 ± 0.17 a	88.47 ± 0.46 b	88.15 ± 0.58 b

Results are expressed as means ± standard deviation (*n* = 3). Different letters within the same row indicate significant differences (*p* < 0.05); ns = not significantly different (*p* > 0.05). Control = no watermeal, WF1 = 1% watermeal, WF3 = 3% watermeal, WF5 = 5% watermeal.

**Table 6:** Compositions of phenolic acids and flavonoids in watermeal noodles compared to control sample.

Parameter	Control	WF1	WF3	WF5
Phenolic acid content (µg/g db)				
Gallic acid	ND	ND	96.00 ± 0.38 b	100.21 ± 0.83 a
Protocatechuic acid	ND	ND	0.43 ± 0.01 b	3.28 ± 0.06 a
*p*-Hydroxybenzoic acid	ND	ND	ND	ND
Vanillic acid	ND	ND	ND	ND
Caffeic acid	ND	ND	ND	ND
Syringic acid	ND	ND	ND	ND
Vanillin	ND	ND	ND	ND
*p*-Coumaric acid	ND	0.51 ± 0.02 c	2.25 ± 0.02 b	4.28 ± 0.12 a
Ferulic acid	1.25 ± 0.01 d	7.34 ± 0.02 c	15.77 ± 0.45 b	22.08 ± 1.78 a
Sinapic acid	2.74 ± 0.08 c	1.8 ± 0.03 c	8.48 ± 0.40 b	14.24 ± 1.63 a
Cinamic acid	ND	1.99 ± 0.08 c	6.50 ± 0.14 b	9.89 ± 0.11 a
Genistic acid	ND	ND	ND	ND
Total	3.99 ± 0.09 d	11.64 ± 0.15 c	129.43 ± 1.01 b	153.98 ± 3.64 a
Flavonoid content (µg/g db)				
Rutin	ND	8.14 ± 0.6 b	30.93 ± 0.61 a	33.76 ± 1.23 a
Catechin	ND	ND	ND	ND
Quercetin	59.91 ± 2.31 b	69.04 ± 0.52 ab	69.57 ± 1.98 ab	73.51 ± 1.3 a
Apigenin	24.74 ± 1.46 c	29.74 ± 0.48 c	85.78 ± 3.16 b	140.04 ± 7.34 a
Karmferal	20.39 ± 1.34 c	21.12 ± 0.3 c	27.33 ± 0.08 b	33.04 ± 2.36 a
Total	105.21 ± 5.11 d	128.04 ± 3.50 c	213.61 ± 5.24 b	280.35 ± 8.92 a

Results are expressed as means ± standard deviation (*n* = 3). Different letters within the same row indicate significant differences (*p* < 0.05). Control = no watermeal, WF1 = 1% watermeal, WF3 = 3% watermeal, WF5 = 5% watermeal, ND = not detected.

**Table 7:** *In vitro* protein digestibility and total sulfhydryl content of watermeal noodles compared to control sample.

Sample	*In Vitro* Protein Digestibility (%)	Total Sulfhydryl (μmol/g)
Control	66.68 ± 0.36 d	2.24 ± 0.01 c
WF1	70.32 ± 0.19 c	2.25 ± 0.02 c
WF3	76.66 ± 0.31 b	2.97 ± 0.07 b
WF5	81.51 ± 0.68 a	4.44 ± 0.00 a

Results are expressed as means ± standard deviation (*n* = 3). Different letters within the same column indicate significant differences (*p* < 0.05). Control = no watermeal, WF1 = 1% watermeal, WF3 = 3% watermeal, WF5 = 5% watermeal.

**Table 8:** *In vitro* starch digestibility of uncooked and cooked watermeal noodles compared to control sample.

Sample	Uncooked				Cooked			
	RDS (%)	SDS (%)	RS (%)	eGI	RDS (%)	SDS (%)	RS (%)	eGI
Control	41.73 ± 0.16 a	16.15 ± 0.66 c	42.12 ± 0.67 b	60.58 ± 0.19 a	51.29 ± 0.54 a	21.58 ±0.39 a	27.13 ± 0.80 d	69.69 ± 0.43 a
WF1	40.77 ± 0.16 b	16.19 ± 0.08 c	43.04 ± 0.08 ab	59.84 ± 0.12 b	50.59 ± 0.41 b	19.21 ± 0.34 b	30.21 ± 0.26 c	68.23 ± 0.37 b
WF3	38.76 ± 0.59 c	17.44 ± 0.51 b	43.8 ± 0.40 a	59.00 ± 0.90 c	46.52 ± 0.40 c	21.72 ± 0.30 a	31.76 ± 0.61 b	67.43 ± 0.29 c
WF5	37.27 ± 0.56 d	19.14 ± 0.47 a	43.59 ± 0.90 a	58.34 ± 0.23 d	44.71 ± 0.29 d	19.96 ± 0.93 b	35.33 ± 0.66 a	65.19 ± 0.04 d

Values are expressed as mean ± standard deviation (*n* = 3), and values with different letters in the same column are considered significantly different (*p* < 0.05). RDS, rapidly digestible starch; SDS, slowly digestible starch; RS, resistant starch; eGI, estimated glycemic index. Control = no watermeal, WF1 = 1% watermeal, WF3 = 3% watermeal, WF5 = 5% watermeal.

**Table 9:** The sensory acceptability of watermeal noodles compared to the control sample.

Sample	Appearance	Color	Odor ^ns^	Taste	Texture	Overall Liking
Control	7.40 ± 1.16 a	7.16 ± 1.49 a	6.70 ± 1.49	7.23 ± 1.52 a	7.26 ± 1.26 a	6.87 ± 1.25 ab
WF1	6.56 ± 1.74 b	6.36 ± 1.21 b	6.93 ± 0.91	6.90 ± 1.54 bc	7.50 ± 1.20 a	7.30 ± 0.99 a
WF3	6.23 ± 1.63 b	6.00 ± 1.51 b	6.63 ± 1.10	6.27 ± 1.51 c	5.63 ± 1.71 b	6.63 ± 1.73 b
WF5	6.06 ± 1.86 b	5.97 ± 1.79 b	6.63 ± 1.25	5.40 ± 1.65 d	5.60 ± 1.81 b	6.36 ± 1.63 b

Results are expressed as means ± standard deviation (*n* = 30). Different letters within the same column indicate significant differences (*p* < 0.05); ns = not significantly different (*p* > 0.05). Control = no watermeal, WF1 = 1% watermeal, WF3 = 3% watermeal, WF5 = 5% watermeal.

## Data Availability

The original contributions presented in this study are included in the article. Further inquiries can be directed to the corresponding author.
